# Using combined *Lactobacillus* and quorum quenching enzyme supplementation as an antibiotic alternative to improve broiler growth performance, antioxidative status, immune response, and gut microbiota

**DOI:** 10.1016/j.psj.2022.101997

**Published:** 2022-06-10

**Authors:** X.X. Sun, D.D Chen, S.Q. Deng, G.M. Zhang, X. Peng, R.N. SA

**Affiliations:** ⁎ The State Key Laboratory of Animal Nutrition, Institute of Animal Sciences, Chinese Academy of Agricultural Sciences, Beijing 100193, China; † Tianjin Biofeed Technology Co., Ltd, TianJin 301906, China

**Keywords:** broiler, *Lactobacillus*, quorum quenching enzyme, growth performance, microbiota

## Abstract

To seek viable alternatives to antibiotics, we determined the combinatorial effects of *Lactobacillus* and a quorum quenching enzyme (**QQE**) on broiler growth performance, antioxidant capacity, immune responses, and cecal microbial populations. In total, 360 one-day-old male broilers (Ross 308) were randomly allotted to 3 dietary treatments, with 12 replicate pens/treatment and 10 birds/replicate pen. Dietary treatments lasted 42 d and comprised: corn-soybean meal basal diet (control group, **CON**); control plus antibiotic growth promoter supplement group (**AGP**); and control plus *Lactobacillus* and QQE supplement group (**LQ**). Dietary LQ supplementation significantly increased final body weight (**BW**) and average daily gain (**ADG**) when compared with CON and AGP groups between 22 and 42 d and 1 to 42 d (*P* < 0.05). No significant differences were observed for serum superoxide dismutase (**SOD**), glutathione peroxidase (**GSH-Px**), and malondialdehyde (**MDA**) levels between treatments (*P* > 0.05). A higher concentration of total antioxidant capacity (**T-AOC**) was observed on d 42 in the LQ group (*P* = 0.06). Feeding LQ significantly increased serum immunoglobulins (IgA and IgG) levels when compared with other treatments (*P* < 0.05). A statistical trend was also observed for increased cecal butyrate levels (*P* = 0.06) in the LQ group. Bacterial α-diversity was unaffected by dietary treatments (*P* > 0.05). However, from principal component analysis (**PCoA**), the microbial community structure was different between the LQ and AGP groups. Diet supplemented with LQ significantly (*P* < 0.05) decreased the relative abundance of *Synergistota* and *Proteobacteria* and significantly (*P* < 0.05) increased the proportion of *Ruminococcaceae* and *Faecalibacterium*. Thus, supplemental LQ improved growth performance, immune status, and modulated intestinal microbial communities in broilers. We provide a new perceptive on antibiotic substitutes in the poultry industry.

## INTRODUCTION

For many years, antibiotics as feed additives have been used to promote the growth of broiler chickens (Engberg et al., 2000). However, increased antibiotic use has led to the emergence of antibiotic-resistance and antibiotic residues in animal products (Lhermie et al., 2016). Restrictions or total bans on antibiotic additives in feed have gradually been implemented in many countries (Lee et al., 2011; Yadav and Jha, 2019). As a consequence, effective additives need to be generated as alternatives to antibiotics, including feed enzymes, probiotics, prebiotics, and organic acids (Gadde et al., 2017).

*Lactobacillus* as live microorganism, which was administered in diet of animals, has been demonstrated to improve growth performance (Li et al., 2018b; Wang et al., 2021a). Various studies have indicated that *Lactobacillus* could benefit nutrient absorption (Wang et al., 2020), antioxidative capacity (Wu et al., 2019a), anti-inflammatory (Wang et al., 2015), intestinal health, and growth performance of broilers (He et al., 2019; Wu et al., 2019b). Wu et al. (2021) reported that dietary *Lactobacillus acidophilus* (10 × 10^8^ CFU/kg) supplementation during d 1 to 21 consistently elevated body weight (**BW**), average daily gain (**ADG**), average daily feed intake (**ADFI**), and jejunum and ileum villus height to crypt depth ratios at 21 d in the presence/absence of an *Escherichia coli* challenge. Lokapirnasari et al. (2019) determined that the addition of 0.25% *Lactobacillus casei* and 0.5% *Bifidobacterium s*pp. improved growth performance and egg production in laying hens. A few studies found that growth promoting effect was not significant (Fatufe and Matanmi, 2008), probably due to the kinds of bacterial species/strains, the supplementation methods, processing technologies, different supplementation levels, and environmental systems.

Acyl homoserine lactones (**AHLs**) are important signal molecules in quorum sensing systems of most gram-negative bacteria (Papenfort and Bassler, 2016). Many pathogenic behaviors of gram-negative bacteria, such as host adhesion, sporulation, exoenzyme production, toxin secretion, biofilm formation, siderophores, and pigment production, are regulated by AHL-mediated Quorum Sensing (**QS**) (Perez and Hagen, 2010; Rutherford and Bassler, 2012). Quorum quenching enzymes (**QQE**), which degrade the AHLs, has been successfully used as a novel ecofriendly method to control important gram-negative pathogens in aquaculture (Chu et al., 2011; Torres et al., 2016; Chen et al., 2020; Sikdar and Elias, 2020). Dietary supplementation with quorum quenching Bacillus strains for 35 d, the growth parameters, digestive enzymes activity and survival rate were improved with Asian seabass under normal feeding conditions (Ghanei-Motlagh et al., 2021).

The aim of this study was to evaluate the combinatorial effects of *Lactobacillus* and QQE on broiler growth performance, antioxidant capacity, immune parameters, and gut microbial populations. We provided the research work on the combinatorial effects of probiotics and enzymes on broilers and explored more ecofriendly antibiotic alternatives.

## MATERIALS AND METHODS

### Ethics Approval

Experimental procedures were approved by the Animal Welfare Committee of the Institute of Animal Sciences, Chinese Academy of Agricultural Sciences (Beijing, China). Studies were performed in accordance with the Guidelines for Care and Use of Laboratory Animals of Chinese Academy of Agriculture Sciences.

### Animals, Experimental Design, and Diets

A total of 360 one-day-old Ross 308 broilers, obtained from a commercial hatchery (Shandong Minhe Animal Husbandry Co., Ltd. Shandong, China), were randomly allotted by weight to 1 of 3 treatment in a completely randomized design. Each treatment consisted of 12 replicate pens with 10 chicks each. Three dietary treatments included 1) basal diet (**CON** group); 2) basal diet + 45 mg/kg chlortetracycline (15%) + 10 mg/kg kitasamycin (45%) (**AGP** group); and 3) basal diet + 50 mg/kg *Lactobacillus* (1 × 10^12^ CFU/g) + 500 mg/kg QQE (10,000 IU/g) (**LQ** group). *Lactobacillus* and QQE products were obtained from Tianjin Biofeed Technology Co., Ltd. (Tianjin, China). The chlortetracycline and kitasamycin were used in broiler diets as AGPs to enhance growth and improve feed efficiency before AGPs were banned, and were usually used as a positive control to evaluate alternatives to AGP (Dong et al., 2011; Han et al., 2020; Pirzado et al., 2021; Gao et al., 2022; Song et al., 2022a).

The corn-soybean basal diets were formulated to meet broiler nutrient requirements as recommended by the National Research Council (1994) and without any AGP and enzymes (Table 1). All chicks were raised on wire-floored cages in the present study. Feed and water were provided ad libitum. Chicks were managed according the guidelines suggested by Ross Broiler Management (Aviagen, 2018). The study lasted 42 d; the starter phase was 1 to 21 d and the grower phase was 22 to 42 d.

**Table 1:** Composition and nutrient levels of the basal diet (air-dry base).

Items	Content	
	1 to 21 d of age	22 to 42 d of age
Ingredients (%)		
Corn	57.20	61.32
Soybean meal	32.00	25.00
Corn gluten meal	3.50	5.00
Soybean oil	2.60	4.00
Limestone	1.20	1.30
CaHPO_4_	1.60	1.30
NaCl	0.25	0.20
NaHCO_3_	0.15	0.20
Lys 70%	0.62	0.74
Met 98%	0.14	0.15
Thr 98%	0.14	0.19
Choline 50%	0.10	0.10
Premix^1^	0.50	0.50
Total	100.00	100.00
Nutrient levels^2^		
CP	21.00	19.00
ME (MJ/kg)	12.54	13.16
Ca	0.90	0.85
TP	0.63	0.55
AP	0.37	0.31
Lys	1.28	1.20
Met	0.46	0.46

1 The premix provided the following per kg of diets: VA, 5,000 IU; VD_3_,10,000 IU; VE,75.0 mg; VK_3_, 18.8 mg; VB_1_, 9.8 mg; VB_2_, 28.8 mg; VB_6_, 19.6 mg; VB_12_, 0.1 mg; calcium pantothenate, 58.8 mg; nicotinic acid, 196.0 mg; folic acid, 4.9 mg; biotin, 2.5 mg; Cu (as copper sulfate) 4.0 mg; Fe (as ferrous sulfate) 40.0 mg; Zn (as zinc sulfate), 37.6 mg ; Mn (as manganese sulfate) 50.0 mg; Se (as sodium selenite) 0.2 mg; I (as calcium iodate) 0.2 mg.

2 The nutrient levels are calculated values.

### Growth Performance

Body weight and feed intake per replicates were recorded on d 21 and d 42 after 12 h fast to determined ADG, ADFI, and feed to gain ratio (**F/G**) of broilers for the periods from d 1 to d 21, from d 22 to d 42, and from d 1 to d 42.

### Sample Collection

On d 42, one broiler per replicate was randomly selected for blood collection. Blood was drawn from the wing vein into a 5 mL anticoagulant-free vacuum tube. After resting blood at room temperature for 2 h, serum was generated by centrifuging at 3,000 rpm for 15 min at 4°C, and then was stored at −20°C until required. After that, birds were euthanized by CO_2_ inhalation to allow for intestinal sample collection. Cecal contents (approximately 2–3 g) were aseptically collected into sterile tubes and immediately snap-frozen in liquid nitrogen, and stored at −80°C for intestinal microbial flora and volatile fatty acid (**VFA**) analyses.

### Serum Biochemical Analyses

Glutathione peroxidase (**GSH-Px**), superoxide dismutase (**SOD**), malondialdehyde (**MDA**), and total antioxidant capacity (**T-AOC**) serum levels were measured using a Unico7200 ultraviolet-visible spectrometer (Unico Co. Ltd, Shanghai, China) following kit instructions (Nanjing Jiancheng Bioengineering Institute, Nanjing, China).

Serum immunoglobulins (**IgG, IgM**, and **IgA**) were measured using enzyme-linked immunosorbent assay (**ELISA**) (Zhongshang Boao Biotechnology Co. Ltd. Shanghai, China) according to manufacturer's instructions. Cytokine (tumor necrosis factor-α [**TNF-α**], interferon-γ [**IFN-γ**], and interleukin-1β [**IL-1β**]) serum levels were ELISA assayed according to kit instructions (Kangjia Hongyuan Biotechnology Co. Ltd. Beijing, China).

### VFA Levels

Approximately 0.07 g broiler cecal digesta samples were thoroughly mixed with 1.5 mL distilled water. After centrifugation (10,000 rpm for 10 min), 1.35 mL supernatant was mixed with 0.15 mL 25% (w/v) metaphosphoric acid solution at 4°C for 4 h in a shaded environment. The mixture was then centrifuged at 12,000 rpm for 10 min at 4°C. The supernatant was used for VFA (acetate, propionate, isobutyrate, butyrate, isovalerate, and valerate) composition analysis using a gas chromatography method according to Hao et al. (2021).

### 16S rRNA-Based Microbiota Analysis

Cecal microbial genomic DNA was extracted using the Fast DNA SPIN for soil kit (MP Biomedicals, Solon, OH). The V3–V4 hyper-variable region of bacterial 16S rRNA was amplified using the primer pair: 338F (5’-ACTCCTACGGGAGGCAG CAG-3’) and 806R (5’-GGACTACHVGGGT WTCTAAT-3’) in an ABI Gene Amp 9700 PCR thermocycler (ABI, CA). After amplification and purification, amplicons were pooled in equimolar amounts and paired-end sequenced on an Illumina MiSeq PE300 platform (Illumina, San Diego, CA). Raw reads were deposited into the National Center for Biotechnology Information Sequence Read Archive database (Accession Number: PRJNA759712). Raw sequences were processed using the Majorbio I-Sanger Cloud Platform and chimeric sequences removed. Then α and β-diversity analyses were performed to investigate differences in species composition between samples. The Kruskal-Wallis H test was used to identify significant differential bacterial taxa in cecal microbial communities.

### Statistical Analysis

Statistical data analysis was conducted using one-way ANOVA with multiple comparisons using Fisher LSD tests (SAS 9.4, Institute, Cary, NC). Pearson correlation analysis was conducted between cecal microbiota (the top 25 relative abundance genus) with growth performance and serum immune parameters of broilers on d 42. The R software (version 3.3.1) was used to graph the data. *P*-values < 0.05 were considered statistically significant, and 0.05 < *P* < 0.10 indicated a tendency for significance.

## RESULTS

### Growth Performance

The effects of LQ in a corn-soybean basal diet on broiler performance at different phases are shown in Table 2. No treatment effects on broiler performance were observed from d 1 to 21 (*P* > 0.05). The ADG in broilers fed LQ was significantly higher than CON and AGP groups from d 22 to 42, and over the entire supplemental period (d 1–42; *P* < 0.05). Additionally, broilers in the LQ group were significantly heavier than CON and AGP broilers at d 42 (*P* < 0.05). The ADFI in the LQ group showed an improved tendency between d 22 and 42 (*P* = 0.08). No statistical differences were observed for FCR among groups at any treatment phases (*P* > 0.05).

**Table 2:** Effects of dietary supplementation of LQ on growth performance in broilers.^1^

Items^2^	Treatment^3^			SEM	*P* value
	CON	AGP	LQ		
D 1 BW (g)	39.96	39.89	39.73	0.69	0.97
D 21 BW (g)	739.16	764.08	754.01	9.30	0.18
D 42 BW (g)	2,435^b^	2,415^b^	2,563^a^	35	0.01
D 1–21					
ADFI (g/d)	48.55	49.88	49.23	0.77	0.48
ADG (g/d)	34.96	36.21	35.71	0.45	0.16
F/G	1.39	1.38	1.38	0.02	0.85
D 22–42					
ADFI (g/d)	135.58	131.73	139.55	2.35	0.08
ADG (g/d)	77.09^b^	75.04^b^	82.25^a^	1.39	0.03
F/G	1.76	1.76	1.70	0.03	0.33
D 1–42					
ADFI (g/d)	91.55	90.66	94.18	1.60	0.28
ADG (g/d)	57.02^b^	56.55^b^	60.09^a^	0.82	0.01
F/G	1.61	1.61	1.57	0.03	0.58

a,b Values in the same row with different superscripts were significantly different (*P* < 0.05) while with same superscripts were insignificantly different (*P* > 0.05).

1 Values are expressed as means with SEM.

2 Abbreviations: ADG, average daily gain; ADFI, average daily feed intake; BW, body weight; F/G, feed: gain ratio.

3 CON, broilers fed a basal diet; AGP, broilers fed a basal diet supplemented with 45 mg/kg chlortetracycline (15%) and 10 mg/kg kitasamycin (45%); LQ, broilers fed a basal diet supplemented with 50 mg/kg Lactobacillus (1 × 10^12^ CFU/g) and 500 mg/kg quorum quenching enzyme (10,000 IU /g).

### Serum Antioxidant Status

Antioxidant data are given in Table 3. On d 42, no significant effects from dietary treatments were observed for SOD, GSH-Px activity, and MDA levels (*P* > 0.05). T-AOC activity in LQ and AGP groups showed an improved tendency (*P* = 0.06).

**Table 3:** Effects of dietary supplementation of LQ on antioxidant capacity in broilers.^1^

Items^2^	Treatment^3^			SEM	*P* value
	CON	AGP	LQ		
T-AOC (mmol/L)	0.29	0.41	0.42	0.04	0.06
SOD (U/mL)	157.48	172.00	162.06	5.68	0.21
GSH-Px (U/mL)	326.33	349.40	358.46	16.10	0.37
MDA (nmol/mL)	5.06	3.93	4.79	0.46	0.22

1 Values are expressed as means with SEM.

2 Abbreviations: GSH-Px, glutathione peroxidase; MDA, Malondialdehyde; SOD, superoxide dismutase; T-AOC, total antioxidant capacity.

3 CON, broilers fed a basal diet; AGP, broilers fed a basal diet supplemented with 45 mg/kg chlortetracycline (15%) and 10 mg/kg kitasamycin (45%); LQ, broilers fed a basal diet supplemented with 50 mg/kg Lactobacillus (1 × 10^12^ CFU/g) and 500 mg/kg quorum quenching enzyme (10,000 IU/g).

### Immunoglobulin and Cytokine Serum Levels

As shown in Table 4, when compared with CON and AGP groups, LQ significantly increased IgA and IgG levels (*P* < 0.05). TNF-ɑ, IFN-γ, and IL-1β serum levels showed no significant differences among groups (*P* > 0.05).

**Table 4:** Effects of dietary supplementation of LQ on immune status in broilers.^1^

Items^2^	Treatment^3^			SEM	*P* value
	CON	AGP	LQ		
IgA (g/L)	0.80^b^	0.84^b^	1.12^a^	0.07	0.02
IgG (g/L)	5.45^b^	6.10^b^	7.45^a^	0.44	0.02
IgM (g/L)	0.78	0.78	0.75	0.06	0.91
TNF-ɑ (pg/mL)	72.40	69.07	79.10	3.73	0.19
IFN-γ (pg/mL)	66.02	59.04	57.99	3.08	0.17
IL-1β (pg/mL)	33.19	33.07	30.63	1.52	0.43

a,b Values in the same row with different superscripts were significantly different (*P* < 0.05) while with same superscripts were insignificantly different (*P* > 0.05).

1 Values are expressed as means with SEM.

2 Abbreviations: IgA, Immunoglobulin A; IgG, Immunoglobulin G; IgM, Immunoglobulin M; IFN-γ, Interferon-γ; IL-1β, Interleukin-1β; TNF-ɑ, tumor necrosis factor-ɑ.

3 CON, broilers fed a basal diet; AGP, broilers fed a basal diet supplemented with 45 mg/kg chlortetracycline (15%) and 10 mg/kg kitasamycin (45%); LQ, broilers fed a basal diet supplemented with 50 mg/kg Lactobacillus (1 × 10^12^ CFU/g) and 500 mg/kg quorum quenching enzyme (10,000 IU /g).

### Cecal VFA Concentrations

No significant differences in cecum VFA levels were observed among groups (*P* > 0.05; Table 5). When compared with CON and AGP groups, broilers in the LQ groups showed an increased tendency in butyrate levels (*P* = 0.06).

**Table 5:** Effects of dietary supplementation of LQ on VFAs in broilers.^1^

Items	Treatment^2^			SEM	*P* value
	CON	AGP	LQ		
Acetate (μg/g)	95.65	92.42	105.51	5.48	0.24
Propionate (μg/g)	40.86	38.35	45.08	5.59	0.70
Isobutyrate (μg/g)	7.00	7.09	7.00	0.34	0.98
Butyrate (μg/g)	19.75	16.43	23.97	2.06	0.06
Isovalerate (μg/g)	5.40	5.66	5.54	0.29	0.82
Valerate (μg/g)	6.20	6.42	6.74	0.31	0.47

1 Values are expressed as means with SEM.

2 CON, broilers fed a basal diet; AGP, broilers fed a basal diet supplemented with 45 mg/kg chlortetracycline (15%) and 10 mg/kg kitasamycin (45%); LQ, broilers fed a basal diet supplemented with 50 mg/kg Lactobacillus (1 × 10^12^ CFU/g) and 500 mg/kg quorum quenching enzyme (10,000 IU /g).

### Cecal Microbiota Diversity

**Figure 1:**
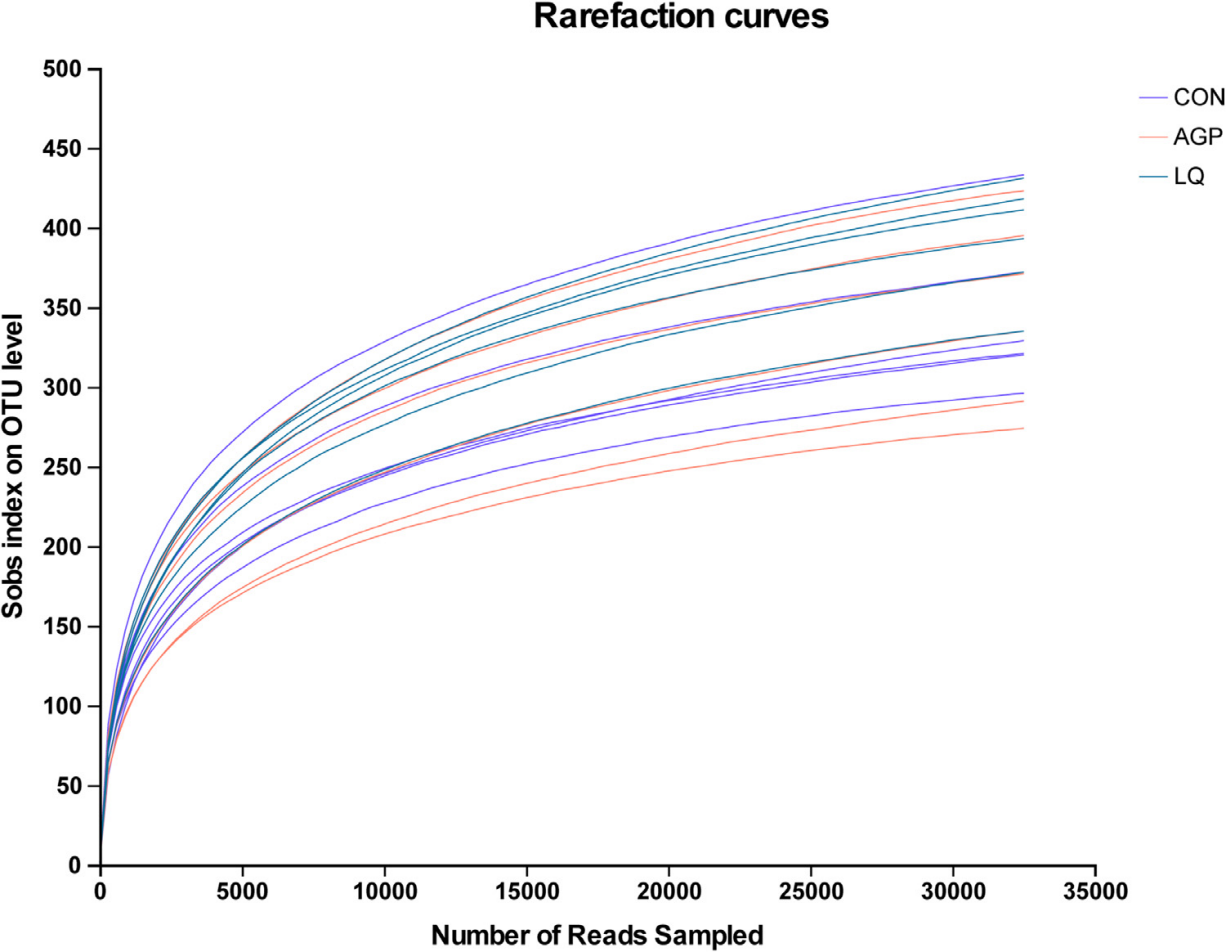
Effects of dietary supplementation of LQ on cecal microbial rarefaction curve of broilers on d 42. CON, broilers fed a basal diet; AGP, broilers fed a basal diet supplemented with 45 mg/kg chlortetracycline (15%) and 10 mg/kg kitasamycin (45%); LQ, broilers fed a basal diet supplemented with 50 mg/kg Lactobacillus (1 × 10^12^ CFU/g) and 500 mg/kg quorum quenching enzyme (10,000 IU /g).

**Figure 2:**
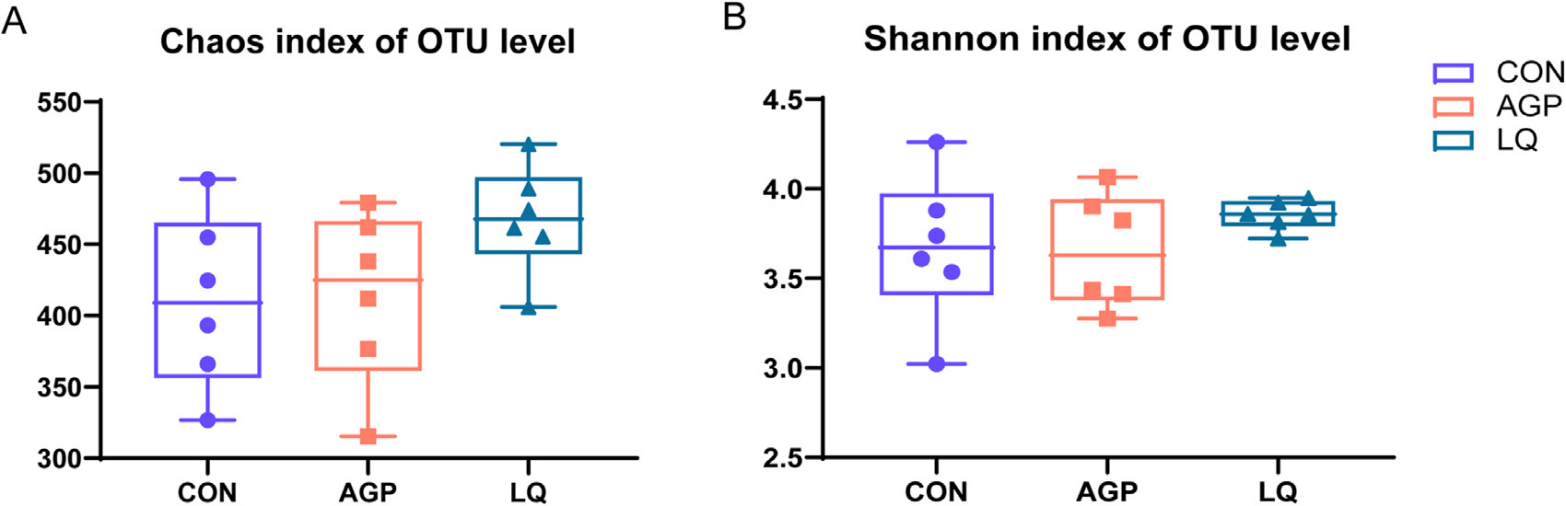
Effects of dietary supplementation of LQ on cecal microbial diversity of broilers on d 42. Significant difference was recorded by *P* < 0.05*. (A) The alpha-diversity of cecal microbiota of Chao index. (B) The alpha-diversity of cecal microbiota of Shannon index.

**Figure 3:**
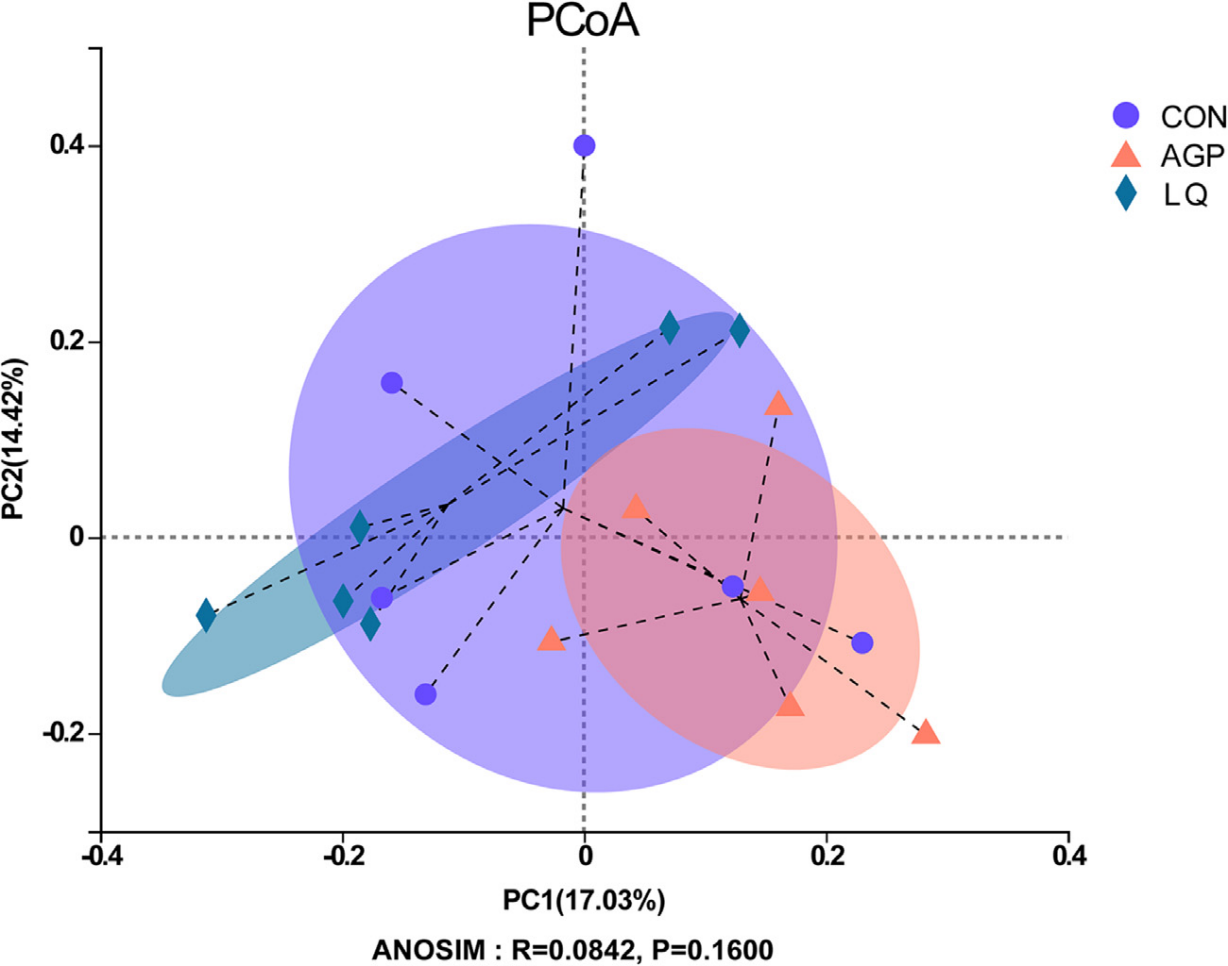
Effects of dietary supplementation of LQ on β-diversity based on bray curtis distance of broilers on d 42.

We conducted 16S rRNA gene sequencing of digesta samples to compare differences in cecal microbiota between groups. The rarefaction curves generated from operational taxonomic units showed that sequencing sufficiently captured most operational units in samples (Figure 1). In terms of α-diversity indices, no significant differences (*P* > 0.05) in Chao and Shannon indices were observed among groups (Figures 2A and 2B). Principal component analysis (**PCoA**) showed that LQ group samples were separately clustered from bacteria in the AGP group (Figure 3).

### Relative Abundance of Cecal Microflora

**Figure 4:**
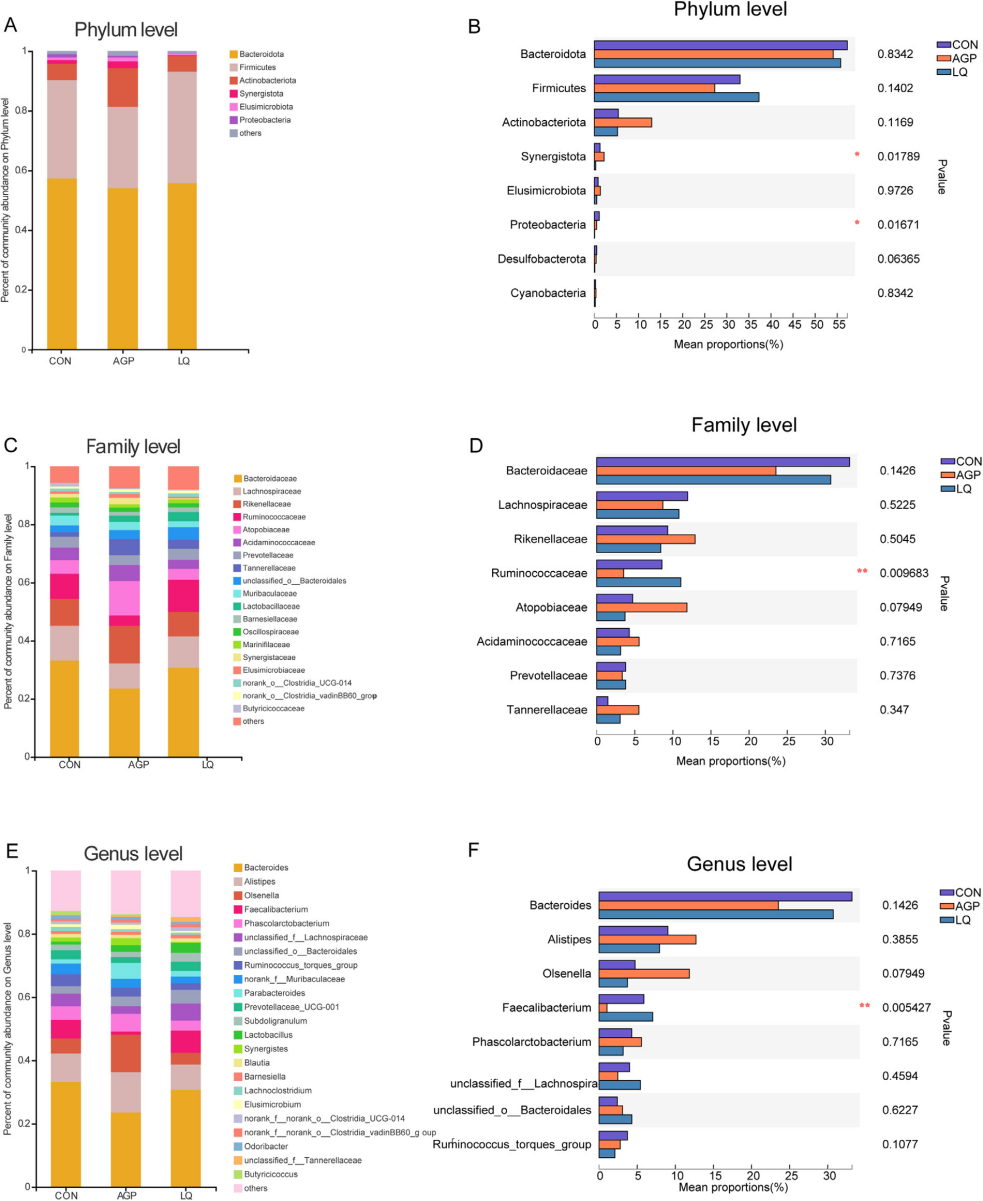
Effects of dietary supplementation of LQ on composition of cecal microbiota and differential species identified at phylum, family and genus level of broilers on day 42. (A, C, E) were microbiota composition at phylum, family and genus level, respectively; (B, D, F) were the differential bacteria at phylum, family and genus level. Significant difference was recorded by 0.01 < *P* ≤ 0.05*, 0.001< *P* ≤ 0.01**.

**Figure 5:**
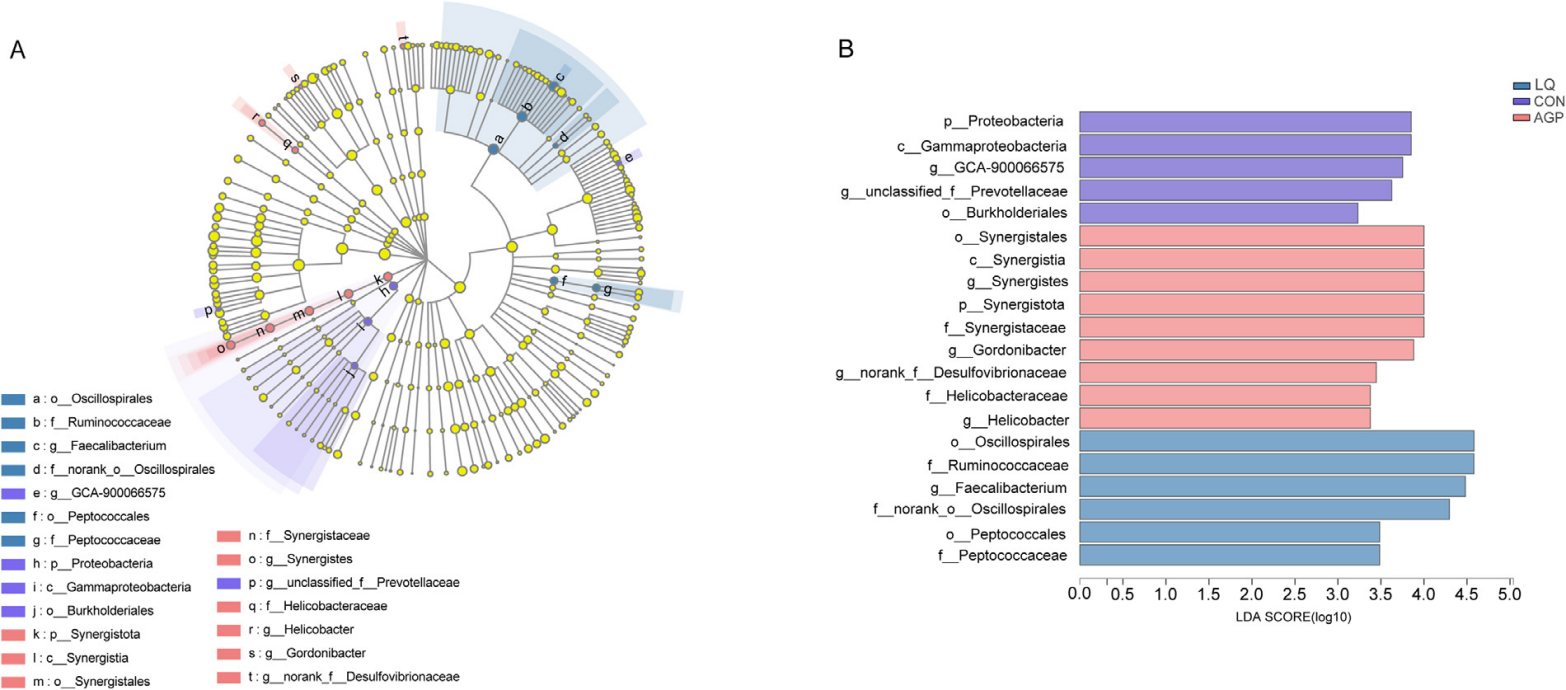
Effects of dietary supplementation of LQ on Linear discriminant analysis effect size (LEfSe) to detect the most significantly abundant cecal microbiota of broilers on d 42 among three groups. (A) Cladoram measured from LEfSe analysis; (B) LDA score generated for differentially abundant microbiota (LDA > 2, *P* < 0.05).

The most abundant phyla in all samples were *Bacteroidetes* and *Firmicutes*, followed by *Actinobacteriota, Synergistota, Elusimicrobiota*, and *Proteobacteria* (Figure 4A). Also, LQ supplementation significantly decreased *Synergistota* and *Proteobacteria* phyla percentages when compared with CON and AGP groups (*P* < 0.05; Figure 4B). At the family level, *Bacteroidaceae, Lachnospiraceae, Rikenellaceae, Ruminococcaceae, Atopobiaceae, Acidaminococcaceae,* and *Prevotellaceae* were the main intestinal flora in all samples (Figure 4C). However, the abundance of the acid-producing bacteria, *Ruminococcaceae* in the cecum of LQ broilers was significantly higher than other groups (*P* < 0.05; Figure 4D). At the genus level, *Bacteroides* and *Alistipes* were the 2 most dominant genera, followed by *Olsenella, Faecalibacterium, Phascolarctobacterium, unclassififiedf-Lachnospiraceae, unclassified-o-Bacteroidales,* and *Ruminococcus-torques-group* (Figure 4E). Additionally, when compared with CON and AGP groups, the beneficial bacteria, *Faecalibacterium* was significantly increased in the LQ group (*P* < 0.05; Figure 4F). Linear discriminant analysis effect-size also showed that *Ruminococcaceae, Oscillospirates, and Faecalibacterium* relative abundance were upregulated in the LQ group, *Synergistes, Helicobacter, and Desulfovibrionaceae* relative abundance were increased in the AGP group, and *Proteobacteria* and *Gammaproteobacteria* relative abundance were relatively higher in the CON group (Figures 5A and 5B).

### Correlation Analysis

**Figure 6:**
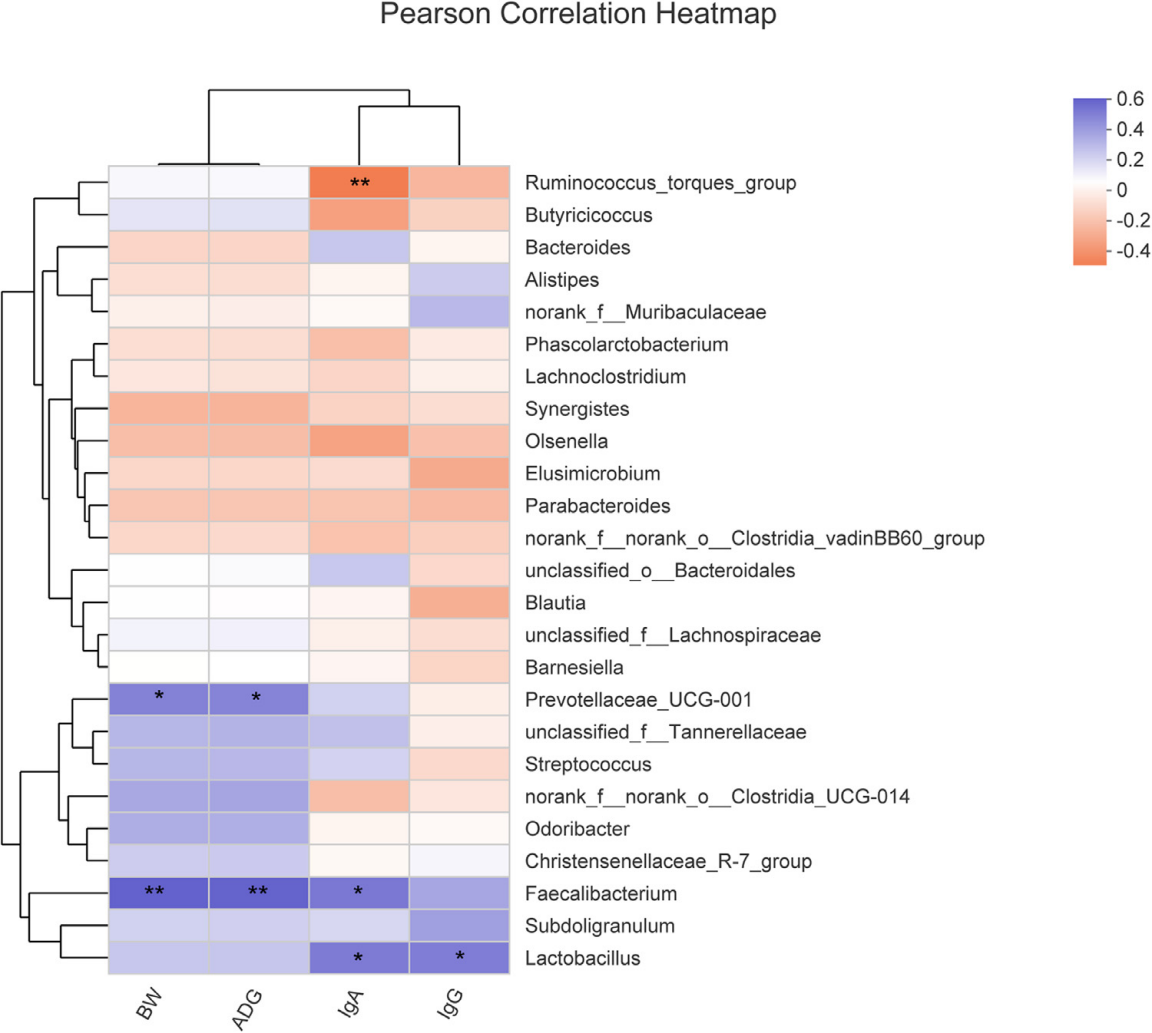
Heatmap of pearson correlation between cecal microbiota (the top 25 relative abundance genus) with growth performance and serum immune parameters of broliers on d 42. Blue suggests a positive correlation, while orange suggests a negative correlation. The intensity of the color indicates the strength of the correlation. The “*” indicates 0.05 < *P* < 0.10, “**” indicates *P* < 0.05.

Pearson analysis was conducted to evaluate the associations between cecal microbiota (the top 25 relative abundance genus) with growth performance and serum immune parameters of broilers (Figure 6). According to the heatmap, the growth performance (d 42 BW and 1–42 d ADG) was positively associated with the abundance of *Faecalibacterium* (*P* < 0.05) and *Prevotellaceae_UCG-001* (*P* = 0.08). The concentration of serum IgA was positively correlated with the abundance of *Faecalibacterium* (*P* = 0.05) and *Lactobacillus* (*P* = 0.06), while negatively correlated with the abundance of *Ruminococcus_torques_group* (*P* < 0.05). Besides, the positive correlation was found between *Lactobacillus* and serum IgG (*P* = 0.07).

## DISCUSSION

Chlortetracycline and kitasamycinas were commonly used as AGPs in commercial farm in China. Various studies have demonstrated that these AGPs had growth-promoting effect on broilers (Dong et al., 2011; Williams et al., 2018; Han et al., 2020; Pirzado et al., 2021). However, in the present study, the AGPs only had a tendency to increase the BW of broilers on d 21, and had no significant effect on BW of broilers on d 42. The results were supported by Bai et al. (2013) who also reported that chlortetracycline only increased the BW of broilers at 21 day of age. The growth-promoting effect of AGPs was decreased when pigs were reared in a clean environment (Cromwell, 2002). The birds in present study were reared in wire-floored cages with a good sanitary condition, which may weaken the growth-promoting effects. The BW of broilers fed LQ diet was higher than that fed AGPs on d 42. Numerous studies found that supplementing with *Lactococcus* in diets increased the performance of broilers (Panda et al., 2006; Apata, 2008; Forte et al., 2017; Wang et al., 2017). QQE was usually used in aquaculture. Ghanei-Motlagh et al. (2021) found that dietary supplementation with 2 quorum quenching *Bacillus* strains increased the digestive enzymes activity and growth parameters of Asian seabass before infection with V. harveyi. The increase in BW of broilers fed LQ diet may be the joint action of *Lactobacillus* and QQE. However, the effects of QQE on the performance of broilers needs to be further studied.

Previous studies showed that probiotic supplementation exerted beneficial effects, including strengthening intestinal barrier function (Wu et al., 2021), stimulating immune systems (Bai et al., 2013), positively modifying intestinal microbiota (Li et al., 2018a), and improving antioxidant activity (Wang et al., 2021b). Antioxidant status within a host serves as an importance to guard against pathogens and maintain homeostasis (Zhu et al., 2015; Fellenberg and Speisky, 2019). In the study, there was an increasing trend of T-AOC in the LQ group and AGP group. Deraz et al. (2019) also found that supplementing with *Lactococcus lactis* and *Lactobacillus plantarum* in broiler diets led to increasing T-AOC concentration and decrease MDA in serum. The administration of dietary *Lactococcus* has been demonstrated to enhance humoral immunity by increasing the serum concentration of immunoglobulins in broilers (Koenen et al., 2004; Wang et al., 2018) and weaned piglets (Dong et al., 2013), which uninfected with pathogens. We also found that the serum concentration of immunoglobulin in LQ group was increased. Moreover, Pearson correlation analysis showed that the relative abundance of *Faecalibacterium* and *Lactobacillus*, which were highest in LQ group among 3 groups, were positively correlated with the concentration of immunoglobulin in serum.

The gut microbiota plays an important role in maintaining gut health and enhancing growth (Marchesi et al., 2016; Rowland et al., 2018; Michaudel and Sokol, 2020). The present study revealed that the supplementation of AGP and LQ had no effects on alpha diversity of cecal microflora in broilers. However, the PCoA showed that the beta diversity was different between LQ and AGP group, which suggested that the effect of LQ treatment on gut microflora was different from that of AGPs. Furthermore, the majority of cecal bacteria in broilers were *Firmicutes, Bacteroidetes,* and *Proteobacteria*, consistent with previous studies (Choi et al., 2014; Mohd Shaufi et al., 2015). *Proteobacteria* is not only related to human intestinal diseases, but also to extraintestinal diseases. These diseases are sustained by various degree of inflammation, which thus represents a core aspect of *Proteobacteria*-related diseases. An increasing amount of data identifies *Proteobacteria* as a possible microbial signature of disease (reviewed by Rizzatti et al., 2017). The relative abundance of *Proteobacteria* was decreased in LQ group compared with CON and AGP group, which indicated that the LQ may improve the intestinal health of broilers. Yang et al. (2020) found that dietary supplementation with *Lactobacillus* reduced the relative abundance of *Proteobacteria* in cecal content of piglets. Li et al. (2018b) reported that the relative abundance of *Proteobacteria* was reduced in the ileum by supplementation with *L. acidophilus* probiotic in bird feed.

The family *Ruminococcaceae* and genus *Faecalibacterium* are predominant intestinal butyrate-producing bacteria (Zhou et al., 2018; Liao et al., 2020; Lan et al., 2021; Oladokun et al., 2021). Butyrate provides energy to intestinal epithelial cells, and plays a key role in inhibiting inflammation and promoting intestinal development (Vinolo et al., 2011; Ratajczak et al., 2019; Ranjbar, et al., 2021). Therefore, the *Ruminococcaceae* and *Faecalibacterium* have been identified as potentially beneficial microbe (Torok et al., 2011). The abundance of *Ruminococcaceae* showed highly positive correlations with the body weight (Stanley et al., 2016; Dai et al., 2020) and better feed conversion in broilers (Stanley et al., 2012; Biddle et al., 2013).The Pearson correlation analysis in this study showed that the relative abundance of *Faecalibacterium* was positively correlated with the BW and ADG of broilers. LQ supplementation significantly increased the relative abundance of *Ruminococcaceae* and *Faecalibacterium*, may further increased the BW and ADG of broilers. Previous study found that *Lactobacillus* supplementation significantly increased the relative abundance of *Ruminococcaceae* in pigs (Yang et al., 2020)*.* Ghanei-Motlagh et al. (2021) reported that dietary supplementation with 2 quorum quenching *Bacillus* strains increased the number of total aerobic heterotrophic bacteria, decreased the number of *Vibrio* spp. in Asian seabass before they were challenged with pathogen. Disrupting bacterial communication (QS) is considered a promising antiviral approach because it can neutralize the pathogens virulence rather than destroys them (Defoirdt, 2018). QQE can reduce the virulence of pathogenic bacteria. It could reduce the colonization of pathogenic bacteria on the intestinal mucosa of broilers, but it also may reduce the colonization of symbiotic bacteria on the mucosa. The effect of QQE on mucosal adhesion bacteria and intestinal health of broilers need to be further studied.

In conclusion, the supplementation of LQ in diets could reduce the relative abundance of *Proteobacteria*, increase the relative abundance of *Ruminococcaceae* and *Faecalibacteriumin*, tend to increase the content of butyric acid, and finally improved the BW and ADG of broilers, which indicated that LQ may be used as a potential alternative to antibiotics in poultry.
